# Supplementary material for: Multisystem recovery after sport-related concussion in adolescent rugby players: a prospective study protocol
Source: BMJ Open. 2023 Aug 2;13(8):e073677. doi: 10.1136/bmjopen-2023-073677 (PMC10401252; doi:10.1136/bmjopen-2023-073677)
Supplement: Author's manuscript [file bmjopen-2023-073677.draft_revisions.pdf]

BMJ Open is committed to open peer review. As part of this commitment we make the peer review history of every article we publish publicly available.

When an article is published we post the peer reviewers' comments and the authors' responses online. We also post the versions of the paper that were used during peer review. These are the versions that the peer review comments apply to.

The versions of the paper that follow are the versions that were submitted during the peer review process. They are not the versions of record or the final published versions. They should not be cited or distributed as the published version of this manuscript.

BMJ Open is an open access journal and the full, final, typeset and author-corrected version of record of the manuscript is available on our site with no access controls, subscription charges or pay-per-view fees (http://bmjopen.bmj.com).

If you have any questions on BMJ Open's open peer review process please email info.bmjopen@bmj.com

BMJ Open

Multi-system recovery after sports-related concussion in adolescent rugby players: A prospective study.

Journal:	*BMJ Open*
Manuscript ID	bmjopen-2023-073677
Article Type:	Protocol
Date Submitted by the Author:	13-Mar-2023
Complete List of Authors:	McKee, Connor; Ulster University - Belfast Campus,<br>Matthews, Mark; Ulster University<br>Rankin, Alan; Sports Medicine NI, Sports Medicine<br>Bleakley, Chris; Ulster University
Keywords:	Adolescent, SPORTS MEDICINE, Paediatric neurology < PAEDIATRICS

SCHOLARONE™
Manuscripts

Title

Multi-system recovery after sports-related concussion in adolescent rugby players: A prospective study.

Authors:

Connor McKee- Ulster University, Jordanstown (mckee-c46@ulster.ac.uk).

Mark Matthews- Ulster University, Jordanstown (m.matthews@ulster.ac.uk).

Alan Rankin- Sports Medicine NI, Castlereagh (alanrankin@doctors.org.uk).

Chris Bleakley- Ulster University, Jordanstown- (c.bleakley@ulster.ac.uk)- **Corresponding Author.**

Abstract

Background: Sports-related concussion is one of the most common injuries in adolescent rugby players with evidence of prolonged recovery. Concussion is a complex pathophysiological process that can affect a variety of subsystems with multifactorial presentation. Most research on adolescents recovery after concussion focus on neurocognitive functioning and symptom outcomes over the short term. There is a need to explore concussion recovery over time across multiple subsystems in adolescent rugby players.

Methods: This prospective study will utilise sensorimotor and oculomotor outcomes in adolescent male and female rugby players aged 16-18 years. Players will be recruited from school or club rugby teams across the province of Ulster. Baseline assessment will be undertaken at the start of the playing season and will include questionnaires, Quantified Y-balance test and Pupillary Light Reflex. Players who sustain a concussive event will be reassessed on all outcomes at 3, 7, 14, 23, 90, 180 and 365 days post-concussion. For serial outcome data we will examine response curves for each participant, and make comparisons between known groups. We will use logistic regression to explore any association between demographic variables and recovery. The strength of the predictive model will be determined using R2, p-values, Odds Ratios, with 95% confidence intervals (95% CI).

Discussion: Sport-related concussion incidence in rugby union remains high, with adolescent players at greater risk of protracted recovery. This prospective study will assess player recovery from a sports-related concussion across key subsystems to develop an understanding of concussion aetiology in adolescents and inform more evidence-based anticipatory guidance.

Strengths

- Female adolescent rugby players have been overlooked in adolescent concussion research, this study seeks to address this with a large sample of both male and female adolescent rugby players.

For peer review only

- Many studies cease follow-up within 6-weeks post-concussion, this study adopts a prospective design which aims to track recovery of sport-related concussion across a playing season, and for up to 12 months post-concussion.
- This study utilises innovative, portable and relatively low-cost technology, that may be feasible for use in a non-professional, youth sporting environment.

Limitations

- Despite the growing popularity of female rugby in Northern Ireland, there is a limited population of female adolescent rugby players, in particular aged 16-18, to recruit from.
- Access to players at key follow-up dates may be challenging given the limited access to adolescent rugby players during school hours at an important exam stage for this population.

Trial Registration Number: ACTRN12622000931774p

Keywords

Concussion, Recovery, Adolescents.

Word Count

3397

Background

Sport-related concussion is a complex pathophysiological process affecting the brain, induced by traumatic biomechanical forces (1). Paediatric visits to emergency departments (EDs) for concussion and minor head injuries have doubled in the United States in recent years (2). Many concussions occur during exercise, particularly during contact and collision sports such as rugby union, ice hockey and American Football. The rate of concussion in professional rugby union is estimated at 21.5 per 1000 game hours (Rafferty, 2019). Incidence is also high in adolescent rugby and prospective research estimates an incidence of 6.1 concussions per 1000 game hours, which is equivalent to 3 concussions per team per season (3).

Sport-related concussion can affect a variety of subsystems, including oculomotor (4), somatosensory (5), vestibular systems (6), with disturbances to cognition (1), mood (7), and changes to biomarker profile (8). Clinical recovery after a sports-related concussion can typically take between 10 and 14 days in adults (1), but can be protracted for up to 4 weeks in adolescents (9). It is recommended adolescent athletes should undergo a battery of tests post-concussion (1, 10-12), including, but not exclusively limited to the Sports Concussion Assessment Tool (SCAT)(13). Whilst research into the recovery from concussion in adolescents is growing, most research focuses primarily on neurocognitive functioning, subjective and symptom based-outcomes (14-16). Few prospective studies examine

For peer review only

sensorimotor or oculomotor function post-concussion, and the natural progression of recovery for these subsystems remains poorly understood.

The sensorimotor system plays a central role in regulating postural control and balance. Retrospective data suggests that 30% of adolescents presenting with concussion have vestibulo-ocular deficits in the acute post injury period (17). Adolescent players with acute vestibular dysfunction also have worse prognosis (18), with a fourfold greater risk of developing post-concussion syndrome (19). Clinical assessment of sensorimotor function is based on balance tests such as balance error scoring system (BESS), dynamic tasks like the Y balance test, or a 3-m walk test. However, these traditional tests are scored subjectively, and lack the responsiveness needed to detect small but important deficits in sensorimotor performance. Adding wearable sensor technology to traditional balance tests can improve their accuracy, allowing researchers to quantify subclinical balance deficits. Previous research has shown that sensor-based instrumentation of the Y balance test provides a reliable and valid measure of movement control (20, 21), and is more responsive than the traditional reach distance scoring method (22). In a study of adolescent rugby union athletes aged 14-18yrs, those with a history of concussion had poorer dynamic balance (high sample entropy [Sen]) during Y-balance tests compared with healthy control (23). There is evidence of quantified Y-balance tests predictive validity, and elite male rugby union players presenting with greater irregularity in their Y balance test movement control were 3 times more likely to sustain a concussion than the players who possessed more regular Y balance test movement control (22).

Oculomotor dysfunction commonly occurs following sports-related concussion (18). This can manifest through symptoms of blurred vision and sensitivity to light. Abnormalities have been observed in the Pupillary Light Response (PLR) following concussion (24). PLR is underpinned by a complex autonomic function, whereby pupillary constriction is dominated by the parasympathetic pathway, and dilation dominated by the sympathetic system. Traditionally PLR was examined subjectively using a penlight test, where the practitioner shines a light into a patient's eyes; but this lacks diagnostic accuracy and results can be confounded by changes in attention, accommodation, and environmental ambient light. Pupillometry is a diagnostic technology, offering a standardised and objective evaluation of the PLR. It provides a quick, accurate and quantifiable evaluation of the PLR (24) and captures a range of outcome parameters across different phases of PLR response (eg. peak and average dilatation/constriction velocity). Normative PLR values are documented in adults, but these are affected by age (25), and initial data suggest that adolescents (aged 12-18), and particularly males, show greater maximal pupil diameters, slower maximum constriction velocities, and fewer percent constriction (25). These developmental differences make translating the results of adult studies to children difficult. Recently, PLR technology has been able to accurately display meaningful differences between adolescents athletes with a concussion and those without on 8 out of 9 PLR metrics (24), but no longitudinal data has been reported.

Evidence suggests that maladaptive coping strategies such as fear-avoidance behaviours relating to an injury have also emerged as potentially important prognostic factors in predicting prolonged recovery (26). Fear-avoidance behaviours have increased in relevancy to concussion recovery in adolescents (27, 28). It would be plausible fear avoidance and

For peer review only

anxiety behaviours around potential or perceived injuries could be associated with prolonged symptoms. Recent studies have highlighted fear avoidance behaviours were positively correlated with somatic symptoms, emotional distress and negatively associated with quality of life in a study involving adolescent subjects with concussion (29), which supports findings from adult samples (27, 28). There is a need to explore fear avoidance behaviours, anxiety and depressive symptoms further in the recovery from sports-related concussion in adolescents.

Sport-related concussions in adolescents remain a great concern due to the uncertainty surrounding their pathophysiology, the potential moderating effects of cognitive maturation and poorly understood recovery. New technologies (e.g. wearable inertial sensors and pupillometry) can enhance the existing battery of assessment, providing more objective, natural recovery data for key subsystems (oculomotor, sensorimotor). Sex differences exist throughout development but there is a dearth of prospective research involving adolescent females who sustain a sports-related concussion and recovery. There is a need for longitudinal research in sports-related concussion as many neurophysiological deficits persist beyond the point of clinical symptom resolution (30-34).

Aims

The aim of this study is to prospectively study recovery from a sport-related concussion in adolescent male and female rugby players. The objectives of the study are to (i) examine if sensorimotor and oculomotor outcomes are affected by concussion history, sex, and age, (ii) re-assess sensorimotor and oculomotor outcomes and self-reported measures at time points post-concussion over a 12month period.

This study protocol follows the Strengthening the Reporting of Observational Studies in Epidemiology (STROBE) guidelines (35).

Study design

This study comprises a cross sectional and prospective component, with follow up continuing over a single playing season. Ethical approval has been granted from Ulster University Research Ethics Committee.

Participants WERE NOT involved in the design, or conduct, or reporting, or dissemination plans of our research.

Participants

Participants will be recruited from school or club rugby teams across the Province of Ulster. There are more than 30 school teams with male adolescent players, and 25 registered clubs with dedicated female youth teams, registered across Ulster. Many of these sites have previously been active members of the Rugby Injury Surveillance in Ulster Schools (RISUS) and have successfully collaborated in previous studies (3, 23, 36). To be eligible for inclusion participants must be (i) aged between 16 and 18 years (ii) injury free at the time of recruitment, and (iii) currently playing rugby union in the 2022/23 playing season.

For peer review only

Consenting participants will complete a baseline questionnaire covering demographics characteristics (date of birth, sex, weight, height, concussion history). The researcher team will be present throughout to assist with the understanding and completion of the questionnaire.

Outcome measures

The outcome measures will be a range of physical assessments and self-reported questionnaires, including symptoms and psychological measures as these are often involved in sports-related concussion. All outcome measures will be taken at 3, 7, 14, 21 days and 3, 6, and 12 months post-concussive event.

Physical Assessment:

Quanitified Y Balance Test is a modification of the Star Excursion Balance Test (37). A detailed testing protocol has previously published (23). Participants position themselves on a platform with hands firmly on their hips. They are instructed to use their non-stance leg to slide a box forward as far as possible with their foot and return to the starting upright position in 3 defined directions (anterior [ANT], posteromedial [PM], and posterolateral [PL]). Reach distances are recorded to the nearest 0.5 cm. Individuals complete 3 practice trials, followed by 3 recorded trials in a randomized order. A failed attempt is noted when any of the following errors occurred: touching of the foot down on the floor before returning to the starting position; placing the foot on top of the sliding box for balance; and flicking or kicking the sliding box for a better performance or any loss of balance (38). If an individual meets any of the failure criteria, the reach attempt will be discarded and repeated. During all tests, an inertial sensor (Shimmer3; Shimmer Sensing) is mounted at the level of the fourth lumbar vertebra and secured with a custom-made elastic belt to closely match the acceleration of the body’s centre of mass during the Y balance test excursions. Inertial sensor testing will be captured during testing and analysed offline with MATLAB (2018b; MathWorks). Reach distances and the Y Balance test will be normalized in relation to the individuals leg length and the mean of three trials completed on each direction will be obtained. Movement control during the Y balance test will be quantified using the following variables: normalized reach distance and the SEn of the 3 axes of the gyroscope signal (x, y, and z). SEn of the signal of length, N={x1,x2,x3,…,xN}, will be calculated using the following formula:

$$\text{Sample Entropy} = -\log (A / B)$$

Our interpretation of SEn scores is underpinned by key theories in the optimal variability during human motor performance, described by Stergiou & Decker (2011) whereby, a low SEn score indicates more regular and less complex movement control during the Y balance test reach excursions, while a higher SEn score indicates a higher degree of movement irregularity/complexity (39).

Pupillary Light Reflex (PLR) Metrics will be measured in response to a brief, step-input, white light stimulus (154 milliseconds’ duration; 180 microwatts’ power) via a Neuroptics PLR-3000 handheld, infrared, automated, monocular pupillometer (Neuroptics). This device is US

For peer review only

FDA approved and has been used in similar studies of mTBI in adults (40). The pupillometer captures dynamic responses 32 times per second, analyzing a continuous, 5-second, digital video of the pupillary response to light. The metrics quantified by the device software include: maximum pupil diameter (steady-state pupil size before the light stimulus); minimum pupil diameter (pupil size after maximum constriction in response to the light stimulus); percentage pupil constriction; latency (time to maximum constriction in response to the light stimulus); peak and average constriction velocity; peak and average dilation velocity; and T75 (time for pupil redilation from minimum diameter to 75% maximum diameter). Peak dilation velocity, will be calculated from automated slope-based measures obtained by the pupillometer (Microsoft Excel 2016; Microsoft Corporation).

Trained research staff will conduct PLR assessments in an environment with a room illumination of approximately 350 lux (moderate photopic viewing conditions). Athletes will focus on a 3-m distance target with the non-tested eye for ocular fixation and accommodation during a 5-second measurement period. Monocular measurements are to be repeated at least 3 times for each eye, alternating 1-minute time intervals to allow rapid visual light adaptation, to obtain 2 to 3 artifact-free responses per eye. The combined mean of each pupillometry metric is then calculated for at least 2 assessments without artifacts, defined as blinks or eye movements occurring within the first 3 seconds of the response.

Self-reported questionnaires:

The Post-Concussion Symptom Scale (PCSS) consists of 22 questions that relate to post-concussive symptoms. Users are asked to rate each symptom according to a 7-point likert scale ranging from 0-6. Higher scores indicate a higher severity of post-concussive symptoms. The greatest possible score is 132 and the lowest possible score is 0. The scale has a high internal consistency (41) in healthy and concussed adolescents, its construct validity has been established (42) and it can accurately discriminate between concussed and non-concussed athletes (41).

The Paediatric Fear Avoidance Behaviour after Traumatic Brain Injury Questionnaire (PFAB-TBI) is a 16-item measure designed to assess fear avoidance following paediatric concussion developed based on FAB-TBI. The FAB-TBI was developed to assess fear avoidance behaviour post-concussion in adults. Items are related to avoidance of activities (e.g. work) due to fear of symptom consequences or symptom exacerbation (e.g. worsened headache). For the PFAB-TBI self-report item content will remain the same with the exception of two items. Item four will be changed from “My work might harm my brain” to “My schoolwork might hurt my brain” and item five will be changed from, “I should not do my normal work with my present symptoms” to “I should not do my normal schoolwork with my present symptoms.” The PFAB– TBI asks respondents to rate agreement on statements related to avoidance behaviour over the past month from 0 (strongly disagree) to 3 (strongly agree). A total sum score will be used in the current study, where higher scores indicate greater fear avoidance behaviour (possible range 0–48). Change scores will be calculated across testing dates (29).

The general anxiety disorder (GAD-7) is a seven-item questionnaire that covers questions about possible problems the respondent may have been bothered by over the previous 2

For peer review only

weeks. Each question has four possible responses from “not at all” to “nearly every day” and scored 0 to 3 respectively. The GAD-7 has reported good reliability, as well as criterion, construct, factorial, and procedural validity (43).

The Patient Health Questionnaire (PHQ-9) is a self-reported questionnaire covering nine questions about experienced depressive symptoms. Questions cover interest in things, feelings of hopelessness, sleep, fatigue, appetite, concentration and thoughts. Each question is scored from a "0" (not at all) to "3" (nearly every day) and has been validated for primary care use (44).

The Sport-related concussion clinical profiling questionnaire consisting of 29 questions covering possible symptoms, and severity of symptoms a player may have experienced over the past 24 hours. Each question has a four-point scale ranging from no symptoms experienced to severe, and scored 0 – 3 respectively for each question. The questionnaire explores five possible clinical profiles including cognitive/fatigue, vestibular, ocular, migraine, and anxiety/mood and two modifiers, sleep and neck (45).

Sport-related Concussion events

Sport-related concussion prevalence will be recorded over a single playing season (2022/23). Sport-related concussion definition will follow the criteria set out by the Zurich Consensus Statement on Concussion in Sport (1) i.e. a direct blow to the head, face, neck or elsewhere on the body with an impulsive force transmitted to the head, resulting in one or more of the symptoms in one or more of the following clinical domains (which may or may not have involved loss of consciousness): somatic, cognitive, emotional/behavioural; sleep disturbance. All sport-related concussions must be verified by a medical professional within 48 hours of the injury. When a participant is diagnosed with a concussion, the designated person at each school/club (data champion), will be responsible for contacting the primary researcher (CMcK). The primary researcher (CMcK) will maintain weekly contact with the data champion, sending study reminders via text message or a follow-up phone call. The following information will be inputted for each concussion: date of injury, reporter/person making the diagnosis, site where diagnosis was made, cause of injury (phase of play, direct vs indirect), time to first symptoms, nature of first symptoms.

In the event of a sport-related concussion, all outcome measures will be reassessed by the primary researcher after 3, 7, 14 and 21 days, and 3, 6 and 12 months. Dates of return to training and return to play will be self-reported by the participant or coach. Return to play dates will be cross referenced with the official team lists registered with Ulster Rugby. The researchers involved in this study will have no influence on the return to training or return to play decisions; these decisions will remain external to the current project.

Statistical Analysis

All data will be analysed using the Statistical Package of Social Sciences (SPSS) (Version 26; SPSS Inc).

For peer review only

Baseline outcomes (Concussion history, Y Balance, PLRM) will be summarized using means and standard deviations for scale variables and frequencies and percentages for nominal variables. We will use histograms to examine the frequency distributions of scale variables, and where normality is assumed, we will use independent t-tests to compare baseline data (PCSS, Y Balance, PLRM), with players categorised into two groups based on self-reported history of concussion in the last 12 months (no concussion in the last 12 months or concussion within the last 12 months). The threshold for statistical significance will $P < .01$ for all tests. We will also present mean differences (95% confidence intervals) between groups for scale outcome data.

Sport-related concussions occurring throughout the 2022/23 playing season will be summarized using counts (%). Time to sport-related concussion event will be plotted using survival curves, with participants censored at the end of the playing season. Serial outcome data (PCSS, Y Balance, PLRM, PFAB-TBI) will be measured at 72 hours, 7 days, 14 days, 23 days, 90 days, 180 days and 365 days post-concussion; we will initially determine the number (%) of participants that had returned to baseline at each time point. We will also examine the growth/response curves for each participant, as well as compare curves between known groups such as sex, concussion history etc. As the shape of the time-response curve is uncertain, and may vary across each outcome variable, we will calculate summary measures for each participant based on either the peak value, the time to return to baseline, or the area under the curve. Area under the curve will be calculated using the trapezium rule (by adding the areas under the graph between each pair of consecutive observations). The most suitable summary measure will be used in the calculation of a median score (interquartile ranges) for each outcome variable. Between group comparisons will also be made, comparing curves across two known groups e.g. males/females, history of concussion/no history, age.

In an exploratory analysis, we will use logistic regression to examine the association between demographic variables (age, sex, previous injury) and recovery at 23 day follow up. We will run separate models for each outcome measure (PCSS, Y Balance, PLRM, PFAB-TBI). Our previous research (3) found that 90% of adolescent rugby players return to sport at 23 days post-concussion; this is also the minimal convalesce for adolescent players post-concussion, based on Irish Rugby Football Union guidance. Full recovery will be defined as a return to baseline (pre-season) values for each respective outcome. Initially we will conduct a series of univariate analyses to determine whether any predictor variables are associated with recovery. Correlations among predictor variables will be calculated to screen for any strong co-linearity ($r>0.8$). Predictors demonstrating a p-value less than 0.10 on univariate testing will be entered into a multiple logistic regression analysis. The strength of the predictive model will be determined using R2, p-values, Odds Ratios, with 95% confidence intervals (95% CI).

Sample size

We aim to enrol 250 participants at baseline. This figure allows for a 20% loss to follow-up over the entire study and assumes a 10% concussion prevalence over a single playing season (ref). This will generate recovery data from n=20 participants presenting with a new

For peer review only

concussion. Recruitment will be stratified to include equal numbers of male and female participants (n=125 male; n=125 female).

A sample of 250 participants would also provide sufficient power for detect baseline difference in sensorimotor function. Our previous research (23) had a medium effect difference SMD 0.5, between adolescents with a history of concussion to those with no history, for sensorimotor function with standard deviation of 0.22 units (SEn). Based on an alpha level of 1%, n=180 participants would provide 90% power for detecting a medium effect difference in SEn between groups at baseline.

Discussion

The primary aim of this study is to study recovery from a sport-related concussion in adolescent male and female rugby players and whether this is affected by concussion history, sex, and age. The ability to confidently diagnose a concussion and monitor its resolution in adolescents has proven somewhat challenging. For an initial sport-related concussion diagnosis in adolescents, no single, gold-standard test exists (46), and even widely-accepted tests have questionable reliability, such as the mBESS, with which groups have been found to have high variability and large number of errors (47). Multiple areas of SRC sign and symptom endorsement should be evaluated, which requires an individualized approach to each athlete and injury (46). Given the absence of a diagnostic test or biomarker for concussion in adolescents, the current cornerstone of concussion diagnosis is confirming the presence of a constellation of signs and symptoms, via multiple tests after an individual has experienced a hit to the head or body although existing evidence is insufficient to determine the best combination of measures (48).

A mix of quantitative and qualitative metrics have been chosen to identify and track recovery from concussion including several self-report measures. Despite the documented importance of reporting concussive symptoms, research has suggested that up to half of concussions in adolescent football players are unreported and therefor undiagnosed (49). The mixed-methods approach employed in the proposed research may allow for an interesting view of whether adolescent rugby players under-report symptoms when quantitatively measured metrics are still elevated.

Concussion incidence in rugby union remains high, with adolescent players at greater risk of protracted recovery (9). Previous studies tracking recovery post-concussion in adolescents have focused primarily on neurocognitive functioning and subjective-symptom outcomes and a shortened follow-up period (15). This prospective study aims to track recovery and assess subsystems affected by concussion, primarily oculomotor and sensorimotor over the course of 12 months. New technologies (wearable sensor technology and Pupillometry) and a battery of self-reported questionnaires aim to enhance the accuracy and responsiveness of outcome data, and potentially allowing researchers to quantify subclinical deficits to balance and PLR.

Women’s rugby is growing in popularity but there is little evidence available regarding injuries and in particular recovery from concussion (50). Research has highlighted that they experience worse outcomes associated with concussion (51) and are more likely to

For peer review only - http://bmjopen.bmj.com/site/about/guidelines.xhtml

experience a concussion when compared with males (52) and this highlights an area of focus for our research.

This trial protocol aims to optimise methodological quality and report pragmatic clinical findings by addressing key methodological limitations of other studies that have aimed to track recovery of concussion in adolescents. Key strengths of the study include: (1) a large even sample population of adolescent male and female rugby players, (2) participant stratification into pre-determined subgroups based on predetermined criteria (i.e. concussion history), (3) multiple outcome measures to assess a wide array of deficits associated with sport-related concussion and (4) longitudinal follow-up to explore possible persistence of multi-system deficits associated with SRC.

LIST OF ABBREVIATIONS

SCAT-Sport Concussion Assessment Tool

PCSS-Post-Concussion Symptom Scale

SAC-Standardised Assessment of Concussion

m-BESS-Modified Balance Error Scoring System

PLR-Pupillary Light Response

PFAB-TBI-Paediatric Fear Avoidance Behaviour after Traumatic Brain Injury

DECLARATIONS

Ethics Approval and Consent to Participate

Participating in this study poses little to no risk to participants or their parents. All patients and their parents/guardians will provide written informed consent/assent and will have the ability to withdraw at any time without explanation. Participation in the study will not restrict them from receiving the standard diagnosis and management, as determined by treating physicians. We will ensure that data remain secure, and that patient privacy and confidentiality are maintained.

Ethical approval has been granted through the Ulster University ethics committee.

Consent for Publication

N/A

Availability for Data and Materials

All data generated or analysed during this study will be included in this published article [and its supplementary information files].

For peer review only

Competing Interests

The authors declare that they have no competing interests.

Funding

This research is to be completed during a funded PhD by the Department for the Economy at Ulster University. The funding body will have no impact on the study design, data collection, analysis, interpretation of data or in writing the manuscript.

Author Contributions

All authors contributed to the concept, design and writing of this protocol.

Acknowledgements

Not applicable.

Authors' Information

N/A

Footnotes

N/A

For peer review only

References

1. McCrory P, Meeuwisse W, Dvořák J, Aubry M, Bailes J, Broglio S, et al. Consensus statement on concussion in sport-the 5(th) international conference on concussion in sport held in Berlin, October 2016. British journal of sports medicine. 2017;51(11):838-47.
2. Macpherson A, Fridman L, Scolnik M, Corallo A, Guttmann A. A population-based study of paediatric emergency department and office visits for concussions from 2003 to 2010. (1205-7088 (Print)).
3. Archbold HA, Rankin AT, Webb M, Nicholas R, Eames NW, Wilson RK, et al. RISUS study: Rugby Injury Surveillance in Ulster Schools. British journal of sports medicine. 2017;51(7):600-6.
4. Lawrence JB, Haider MN, Leddy JJ, Hinds A, Miecznikowski JC, Willer BS. The King-Devick test in an outpatient concussion clinic: Assessing the diagnostic and prognostic value of a vision test in conjunction with exercise testing among acutely concussed adolescents. Journal of the neurological sciences. 2019;398:91-7.
5. Corbin-Berrigan L-A, Gagnon I. Postconcussion Symptoms as a Marker of Delayed Recovery in Children and Youth Who Recently Sustained a Concussion: A Brief Report. Clinical journal of sport medicine. 2017;27(3):325-7.
6. Berkner J, Meehan WP, 3rd, Master CL, Howell DR. Gait and Quiet-Stance Performance Among Adolescents After Concussion-Symptom Resolution. Journal of athletic training. 2017;52(12):1089-95.
7. Caze T, Vásquez D, Moffatt K, Waple K, Hope D. A Prospective Pilot Study of Anxiety Sensitivity and Adolescent Sports-Related Concussion. Archives of clinical neuropsychology. 2020;36(6):930-9.
8. Rhine T, Babcock L, Zhang N, Leach J, Wade SL. Are UCH-L1 and GFAP promising biomarkers for children with mild traumatic brain injury? Brain injury. 2016;30(10):1231-8.
9. Davis GA, Anderson V, Babl FE, Gioia GA, Giza CC, Meehan W, et al. What is the difference in concussion management in children as compared with adults? A systematic review. British journal of sports medicine. 2017;51(12):949-57.
10. Guskiewicz KM, Register-Mihalik JK. Postconcussive impairment differences across a multifaceted concussion assessment protocol. PM & R : the journal of injury, function, and rehabilitation. 2011;3(10 Suppl 2):445.
11. Harmon KG, Clugston JR, Dec K, Hainline B, Herring S, Kane SF, et al. American Medical Society for Sports Medicine position statement on concussion in sport. British journal of sports medicine. 2019;53(4):213-25.
12. Rose SC, Fischer AN, Heyer GL. How long is too long? The lack of consensus regarding the post-concussion syndrome diagnosis. Brain injury. 2015;29(7-8):798-803.
13. Echemendia RJ, Meeuwisse W, McCrory P, Davis GA, Putukian M, Leddy J, et al. The Sport Concussion Assessment Tool 5th Edition (SCAT5): Background and rationale. British journal of sports medicine. 2017;51(11):848-50.
14. Howell DR, Brien MJ, Beasley MA, Mannix RC, Meehan Iii WP. Initial somatic symptoms are associated with prolonged symptom duration following concussion in adolescents. Acta Paediatrica. 2016;105(9):e426-e32.

For peer review only

15. Hang B, Babcock L, Hornung R, Ho M, Pomerantz W. Can Computerized Neuropsychological Testing in the Emergency Department Predict Recovery for Young Athletes With Concussions? Pediatric emergency care. 2015;31(10):688-93.
16. Lau B, Lovell M, Collins M, Pardini J. Neurocognitive and Symptom Predictors of Recovery in High School Athletes. Clinical journal of sport medicine. 2009;19(3):216-21.
17. Ellis MJ, Cordingley DM, Vis S, Reimer KM, Leiter J, Russell K. Clinical predictors of vestibulo-ocular dysfunction in pediatric sports-related concussion. Journal of Neurosurgery Publishing Group (JNSPG); 2017. p. 38.
18. Master C, Master S, Wiebe D, Storey E, Lockyer J, Podolak O, et al. Vision and Vestibular System Dysfunction Predicts Prolonged Concussion Recovery in Children. Clinical journal of sport medicine. 2018;28(2):139-45.
19. Ellis MJ, Cordingley D, Vis S, Reimer K, Leiter J, Russell K. Vestibulo-ocular dysfunction in pediatric sports-related concussion. Journal of Neurosurgery Publishing Group (JNSPG); 2015. p. 248.
20. Powers KC, Kalmar JM, Cinelli ME. Recovery of static stability following a concussion. Gait & posture. 2014;39(1):611-4.
21. Broglio SP, Macciocchi SN, Ferrara MS. Sensitivity of the concussion assessment battery. Neurosurgery. 2007;60(6):1050-8.
22. Johnston W, O'Reilly M, Duignan C, Liston M, McLoughlin R, Coughlan GF, et al. Association of Dynamic Balance With Sports-Related Concussion: A Prospective Cohort Study. The American Journal of Sports Medicine. 2019;47(1):197-205.
23. Matthews M, Johnston W, Bleakley CM, Davies RJ, Rankin AT, Webb M, et al. Concussion History and Balance Performance in Adolescent Rugby Union Players. The American Journal of Sports Medicine. 2021;49(5):1348-54.
24. Master CL, Podolak OE, Ciuffreda KJ, Metzger KB, Joshi NR, McDonald CC, et al. Utility of Pupillary Light Reflex Metrics as a Physiologic Biomarker for Adolescent Sport-Related Concussion. JAMA ophthalmology. 2020;138(11):1135-41.
25. Boev AN, Fountas KN, Karampelas I, Boev C, Machinis TG, Feltes C, et al. Quantitative pupillometry: normative data in healthy pediatric volunteers. Journal of neurosurgery. 2005;103(6 Suppl):496-500.
26. Woodrome SE, Yeates KO, Taylor HG, Rusin J, Bangert B, Dietrich A, et al. Coping strategies as a predictor of post-concussive symptoms in children with mild traumatic brain injury versus mild orthopedic injury. Journal of the International Neuropsychological Society : JINS. 2011;17(2):317-26.
27. Silverberg ND, Panenka WJ, Iverson GL. Fear Avoidance and Clinical Outcomes from Mild Traumatic Brain Injury. Journal of neurotrauma. 2018;35(16):1864-73.
28. Wijenberg MLM, Stapert SZ, Verbunt JA, Ponsford JL, Van Heugten CM. Does the fear avoidance model explain persistent symptoms after traumatic brain injury? Brain injury. 2017;31(12):1597-604.
29. Cairncross M, Brooks BL, Virani S, Silverberg ND. Fear avoidance behavior in youth with poor recovery from concussion: measurement properties and correlates of a new scale. Child neuropsychology : a journal on normal and abnormal development in childhood and adolescence. 2021;27(7):911-21.
30. Mutch WAC, Ellis MJ, Ryner LN, Morissette MP, Pries PJ, Dufault B, et al. Longitudinal Brain Magnetic Resonance Imaging CO2 Stress Testing in Individual Adolescent Sports-Related Concussion Patients: A Pilot Study. Frontiers in neurology. 2016;7:107.

For peer review only

31. McGuine TA, Pfaller A, Hetzel S, Broglio SP, Hammer E. A Prospective Study of Concussions and Health Outcomes in High School Football Players. Journal of athletic training. 2020;55(10):1013-9.
32. Howell D, Myer G, Brilliant A, Barber Foss K, Meehan W. Quantitative Multimodal Assessment of Concussion Recovery in Youth Athletes. Clinical journal of sport medicine. 2019;31(2):133-8.
33. Nolin P, Stipanicic A, Henry M, Joyal CC, Allain P. Virtual reality as a screening tool for sports concussion in adolescents. Brain injury. 2012;26(13-14):1564-73.
34. Kelty-Stephen DG, Qureshi Ahmad M, Stirling L. Use of a Tracing Task to Assess Visuomotor Performance for Evidence of Concussion and Recuperation. Psychological assessment. 2015;27(4):1379-87.
35. Cuschieri S. The STROBE guidelines. Saudi journal of anaesthesia. 2019;13(Suppl 1):S31-S4.
36. Archbold P, Rankin A, Webb M, Davies R, Nicholas R, Eames N, et al. Injury patterns in U15 rugby players in Ulster Schools: A Rugby Injury Surveillance (RISUS) study. Translational Sports Medicine. 2021;4.
37. Shaffer SW, Teyhen DS, Lorenson CL, Warren RL, Koreerat CM, Straseske CA, et al. Y-balance test: a reliability study involving multiple raters. Military medicine. 2013;178(11):1264-70.
38. Gribble PA, Hertel J, Plisky P. Using the Star Excursion Balance Test to assess dynamic postural-control deficits and outcomes in lower extremity injury: a literature and systematic review. Journal of athletic training. 2012;47(3):339-57.
39. Stergiou N, Decker LM. Human movement variability, nonlinear dynamics, and pathology: is there a connection? (1872-7646 (Electronic)).
40. Ciuffreda KJ, Joshi NR, Truong JQ. Understanding the effects of mild traumatic brain injury on the pupillary light reflex. Concussion (London, England). 2017;2(3):CNC36-0029. eCollection 2017 Nov.
41. Lovell MR, Iverson GL, Collins MW, Podell K, Johnston KM, Pardini D, et al. Measurement of symptoms following sports-related concussion: reliability and normative data for the post-concussion scale. Applied Neuropsychology. 2006;13(3):166-74.
42. Riegler KE, Guty ET, Arnett PA. Validity of the ImPACT Post-Concussion Symptom Scale (PCSS) Affective Symptom Cluster as a Screener for Depression in Collegiate Athletes. Archives of clinical neuropsychology : the official journal of the National Academy of Neuropsychologists. 2019;34(4):563-74.
43. Spitzer RL, Kroenke K, Williams JBW, Löwe B. A Brief Measure for Assessing Generalized Anxiety Disorder: The GAD-7. Archives of Internal Medicine. 2006;166(10):1092-7.
44. Cameron IM, Crawford JR, Lawton K, Reid IC. Psychometric comparison of PHQ-9 and HADS for measuring depression severity in primary care. The British journal of general practice : the journal of the Royal College of General Practitioners. 2008;58(546):32-6.
45. Kontos A, Trbovich A, Sandel N, Emami K, Collins M. Sport-related Concussion Clinical Profiles: Clinical Characteristics, Targeted Treatments, and Preliminary Evidence. Current Sports Medicine Reports. 2019;18:82-92.
46. McKeithan L, Hibshman N, Yengo-Kahn AM, Solomon GS, Zuckerman SL. Sport-Related Concussion: Evaluation, Treatment, and Future Directions. Medical sciences (Basel, Switzerland). 2019;7(3):44. doi: 10.3390/medsci7030044.

For peer review only

47. Starling AJ, Leong DF, Bogle JM, Vargas BB. Variability of the modified Balance Error Scoring System at baseline using objective and subjective balance measures. Concussion (London, England). 2015;1(1):CNC5.
48. Giza CC, Kutcher JS, Ashwal S, Barth J, Getchius TS, Gioia GA, et al. Summary of evidence-based guideline update: evaluation and management of concussion in sports: report of the Guideline Development Subcommittee of the American Academy of Neurology. Neurology. 2013;80(24):2250-7.
49. McCrea M, Hammeke T, Olsen G, Leo P, Guskiewicz K. Unreported concussion in high school football players: implications for prevention. Clinical journal of sport medicine : official journal of the Canadian Academy of Sport Medicine. 2004;14(1):13-7.
50. Moody JR, Feiss RS, Pangelinan MM. A systematic review of acute concussion assessment selection in research. Brain injury. 2019;33(8):967-73.
51. Baker JG, Leddy JJ, Darling SR, Shucard J, Makdissi M, Willer BS. Gender Differences in Recovery From Sports-Related Concussion in Adolescents. Clinical pediatrics. 2016;55(8):771-5.
52. Mollayeva T, El-Khechen-Richandi G, Colantonio A. Sex & gender considerations in concussion research. Concussion (London, England). 2018;3(1):CNC51-0015. eCollection 2018 Mar.

For peer review only

STROBE Statement—checklist of items that should be included in reports of observational studies

	Item No	Recommendation	Page No
**Title and abstract**	1	(*a*) Indicate the study's design with a commonly used term in the title or the abstract	1
		(*b*) Provide in the abstract an informative and balanced summary of what was done and what was found	1
**Introduction**			
Background/rationale	2	Explain the scientific background and rationale for the investigation being reported	2-4
Objectives	3	State specific objectives, including any prespecified hypotheses	4
**Methods**			
Study design	4	Present key elements of study design early in the paper	4
Setting	5	Describe the setting, locations, and relevant dates, including periods of recruitment, exposure, follow-up, and data collection	4-5
Participants	6	(*a*) *Cohort study*—Give the eligibility criteria, and the sources and methods of selection of participants. Describe methods of follow-up<br>*Case-control study*—Give the eligibility criteria, and the sources and methods of case ascertainment and control selection. Give the rationale for the choice of cases and controls<br>*Cross-sectional study*—Give the eligibility criteria, and the sources and methods of selection of participants	4-8
		(*b*) *Cohort study*—For matched studies, give matching criteria and number of exposed and unexposed<br>*Case-control study*—For matched studies, give matching criteria and the number of controls per case	n/a
Variables	7	Clearly define all outcomes, exposures, predictors, potential confounders, and effect modifiers. Give diagnostic criteria, if applicable	5-9
Data sources/ measurement	8*	For each variable of interest, give sources of data and details of methods of assessment (measurement). Describe comparability of assessment methods if there is more than one group	5-8
Bias	9	Describe any efforts to address potential sources of bias	8
Study size	10	Explain how the study size was arrived at	8-9
Quantitative variables	11	Explain how quantitative variables were handled in the analyses. If applicable, describe which groupings were chosen and why	7-8
Statistical methods	12	(*a*) Describe all statistical methods, including those used to control for confounding	7-9
		(*b*) Describe any methods used to examine subgroups and interactions	7-8
		(*c*) Explain how missing data were addressed	n/a
		(*d*) *Cohort study*—If applicable, explain how loss to follow-up was addressed	8-9

For peer review only - http://bmjopen.bmj.com/site/about/guidelines.xhtml

		Case-control study—If applicable, explain how matching of cases and controls was addressed<br>*Cross-sectional study*—If applicable, describe analytical methods taking account of sampling strategy	
		(*e*) Describe any sensitivity analyses	8-9
**Results**			
Participants	13*	(a) Report numbers of individuals at each stage of study—eg numbers potentially eligible, examined for eligibility, confirmed eligible, included in the study, completing follow-up, and analysed	8-9
		(b) Give reasons for non-participation at each stage	n/a
		(c) Consider use of a flow diagram	n/a
Descriptive data	14*	(a) Give characteristics of study participants (eg demographic, clinical, social) and information on exposures and potential confounders	4-5
		(b) Indicate number of participants with missing data for each variable of interest	n/a
		(c) *Cohort study*—Summarise follow-up time (eg, average and total amount)	7
Outcome data	15*	*Cohort study*—Report numbers of outcome events or summary measures over time	7
		Case-control study—Report numbers in each exposure category, or summary measures of exposure	n/a
		Cross-sectional study—Report numbers of outcome events or summary measures	*n/a*
Main results	16	(*a*) Give unadjusted estimates and, if applicable, confounder-adjusted estimates and their precision (eg, 95% confidence interval). Make clear which confounders were adjusted for and why they were included	7-8
		(*b*) Report category boundaries when continuous variables were categorized	7-8
		(*c*) If relevant, consider translating estimates of relative risk into absolute risk for a meaningful time period	n/a
Other analyses	17	Report other analyses done—eg analyses of subgroups and interactions, and sensitivity analyses	7-8
**Discussion**			
Key results	18	Summarise key results with reference to study objectives	9-10
Limitations	19	Discuss limitations of the study, taking into account sources of potential bias or imprecision. Discuss both direction and magnitude of any potential bias	10
Interpretation	20	Give a cautious overall interpretation of results considering objectives, limitations, multiplicity of analyses, results from similar studies, and other relevant evidence	9-10
Generalisability	21	Discuss the generalisability (external validity) of the study results	9-10
**Other information**			
Funding	22	Give the source of funding and the role of the funders for the present study and, if applicable, for the original study on which the present article is based	11

Continued on next page

For peer review only - http://bmjopen.bmj.com/site/about/guidelines.xhtml

*Give information separately for cases and controls in case-control studies and, if applicable, for exposed and unexposed groups in cohort and cross-sectional studies.

Note: An Explanation and Elaboration article discusses each checklist item and gives methodological background and published examples of transparent reporting. The STROBE checklist is best used in conjunction with this article (freely available on the Web sites of PLoS Medicine at http://www.plosmedicine.org/, Annals of Internal Medicine at http://www.annals.org/, and Epidemiology at http://www.epidem.com/). Information on the STROBE Initiative is available at www.strobe-statement.org.

For peer review only

For peer review only - http://bmjopen.bmj.com/site/about/guidelines.xhtml

BMJ Open

Multi-system recovery after sports-related concussion in adolescent rugby players: A prospective study protocol.

Journal:	*BMJ Open*
Manuscript ID	bmjopen-2023-073677.R1
Article Type:	Protocol
Date Submitted by the Author:	30-Jun-2023
Complete List of Authors:	McKee, Connor; Ulster University - Belfast Campus,<br>Matthews, Mark; Ulster University<br>Rankin, Alan; Sports Medicine NI, Sports Medicine<br>Bleakley, Chris; Ulster University
**Primary Subject Heading**:	Sports and exercise medicine
Secondary Subject Heading:	Paediatrics, Sports and exercise medicine
Keywords:	Adolescent, SPORTS MEDICINE, Paediatric neurology < PAEDIATRICS

SCHOLARONE™ Manuscripts

Title

Multi-system recovery after sports-related concussion in adolescent rugby players: A prospective study protocol.

Authors:

Connor McKee- Ulster University, Jordanstown (mckee-c46@ulster.ac.uk).

Mark Matthews- Ulster University, Jordanstown (m.matthews@ulster.ac.uk).

Alan Rankin- Sports Medicine NI, Castlereagh (alanrankin@doctors.org.uk).

Chris Bleakley- Ulster University, Jordanstown- (c.bleakley@ulster.ac.uk)- **Corresponding Author.**

Abstract

Introduction: Sports-related concussion is one of the most common injuries in adolescent rugby players with evidence of prolonged recovery in some concussed athletes. Concussion is a complex pathophysiological process that can affect a variety of subsystems with multifactorial presentation. Most research on adolescents recovery after concussion focuses on neurocognitive functioning and symptom outcomes over the short term. There is a need to explore concussion recovery over time across multiple subsystems in adolescent rugby players.

Methods and Analysis: This prospective study will utilise sensorimotor and oculomotor outcomes in adolescent male and female rugby players aged 16-18 years. Players will be recruited from school or club rugby teams across the province of Ulster. Baseline assessment will be undertaken at the start of the playing season and will include questionnaires, Quantified Y-balance test and Pupillary Light Reflex. Players who sustain a concussive event will be reassessed on all outcomes at 3, 7, 14, 23, 90, 180 and 365 days post-concussion. For serial outcome data we will examine response curves for each participant, and make comparisons between known groups. We will use logistic regression to explore any association between demographic variables and recovery. The strength of the predictive model will be determined using R^2, p-values and confidence and odds ratios (OR) with 95% confidence intervals (95% CI).

Ethics and Dissemination: Ethical approval has been granted for this study from Ulster University Research Ethics Committee (REC/14/0060). This study will be published in an open access research journal upon completion.

Trial Registration Number: ACTRN12622000931774p

Strengths

For peer review only

- Female adolescent rugby players have been overlooked in adolescent concussion research, this study seeks to address this with a large sample of both male and female adolescent rugby players.
- Many studies cease follow-up within 6-weeks post-concussion, this study adopts a prospective design which aims to track recovery of sport-related concussion for 12 months.
- This study utilises innovative, portable and relatively low-cost technology, that may be feasible for use in a non-professional, youth sporting environment.

Limitations

- Despite the growing popularity of female rugby in Northern Ireland, there is a limited population of female adolescent rugby players, in particular aged 16-18, to recruit from.
- Access to players at key follow-up dates may be challenging given the limited access to adolescent rugby players during school hours at an important exam stage for this population.

Keywords

Female, Ocular, Cognitive, Vestibular, Sensorimotor, Anxiety, Fear, Balance, Pupillary, Light-Reflex

Word Count

4567

Introduction

Sport-related concussion is a complex pathophysiological process affecting the brain induced by traumatic biomechanical forces (1). Paediatric visits to emergency departments (EDs) for concussion and minor head injuries have doubled in the United States in recent years (2). Many concussions occur during contact and collision sports like rugby union. The rate of concussion in professional rugby union is estimated at 21.5 per 1000 game hours (Rafferty, 2019). Incidence is also high in adolescent rugby with estimates of an incidence of 6.1 concussions per 1000 game hours, which is equivalent to 3 concussions per team per season (3).

Sport-related concussion can affect a variety of subsystems, including oculomotor (4), somatosensory (5), vestibular systems (6), with disturbances to cognition (1), mood (7), and biomarker profile (8). Clinical recovery after a sports-related concussion can typically take between 10 and 14 days in adults (1) and up to 4 weeks in adolescents (9). It is recommended adolescent athletes undergo a battery of tests post-concussion (1, 10-12), including, the Sports Concussion Assessment Tool (SCAT)(13). Whilst research into the recovery from concussion in adolescents is growing, most research focuses primarily on neurocognitive functioning, subjective and symptom based-outcomes (14-16). Few

prospective studies examine sensorimotor or oculomotor function post-concussion, and the natural progression of recovery for these subsystems is poorly understood.

The sensorimotor system plays a central role in regulating postural control and balance. Retrospective data suggests that 30% of adolescents presenting with concussion have vestibulo-ocular deficits in the acute post injury period (17). Adolescent players with acute vestibular dysfunction also have worse prognosis (18) with a fourfold greater risk of developing post-concussion syndrome (19). Clinical assessment of sensorimotor function is based on balance tests such as balance error scoring system (BESS), dynamic tasks like the Y balance test, or a 3-m walk test. However, these traditional tests are scored subjectively, and lack the responsiveness needed to detect small but important deficits in sensorimotor performance. Adding wearable sensor technology to traditional balance tests can improve their accuracy, allowing researchers to quantify subclinical balance deficits. Previous research has shown that sensor-based instrumentation of the Y balance test provides a reliable and valid measure of movement control (20, 21), and is more responsive than the traditional reach distance scoring method (22). In a study of adolescent rugby union athletes aged 14-18yrs, those with a history of concussion had poorer dynamic balance (high sample entropy [Sen]) during Y-balance tests compared with non-concussed controls (23). There is evidence of quantified Y-balance tests predictive validity, and elite male rugby union players presenting with greater irregularity in their Y balance test movement control were 3 times more likely to sustain a concussion than the players with more regular Y balance test movement control (22).

Oculomotor dysfunction commonly occurs following sports-related concussion (18). This can manifest through symptoms of blurred vision and sensitivity to light. Abnormalities have been observed in the Pupillary Light Response (PLR) following concussion (24). PLR is underpinned by a complex autonomic function, whereby pupillary constriction is dominated by the parasympathetic pathway, and dilation dominated by the sympathetic system. Traditionally PLR was examined subjectively using a penlight test, where the practitioner shines a light into a patient's eyes; but this lacks diagnostic accuracy and results can be confounded by changes in attention, accommodation, and environmental ambient light. Pupillometry is a diagnostic technology offering a standardised and objective evaluation of the PLR, providing a quick, accurate and quantifiable evaluation of the PLR (24) and capturing a range of outcome parameters across different phases of PLR response (e.g. peak and average dilatation/constriction velocity). Normative PLR values are documented in adults, but these are affected by age (25), and initial data suggest that adolescents (aged 12-18), and particularly males, show greater maximal pupil diameters, slower maximum constriction velocities, and lower percent constriction (25). These developmental differences make translating the results of adult studies to children difficult. Recently, PLR technology has been able to accurately display meaningful differences between adolescents athletes with a concussion and those without on 8 out of 9 PLR metrics (24), but no longitudinal data has been reported.

Evidence suggests that maladaptive coping strategies such as fear-avoidance behaviours relating to an injury have also emerged as potentially important prognostic factors in predicting prolonged recovery (26). Fear-avoidance behaviours have increased in relevancy to concussion recovery in adolescents (27, 28). It would be plausible fear avoidance and

For peer review only

anxiety behaviours around potential or perceived injuries could be associated with prolonged symptoms. Recent studies have highlighted fear avoidance behaviours were positively correlated with somatic symptoms, emotional distress and negatively associated with quality of life in a study involving adolescent subjects with concussion (29), which supports findings from adult samples (27, 28). There is a need to explore fear avoidance behaviours, anxiety and depressive symptoms further in the recovery from sports-related concussion in adolescents.

Sport-related concussions in adolescents remain a great concern due to the uncertainty surrounding their pathophysiology, the potential moderating effects of cognitive maturation and poorly understood recovery. New technologies (e.g. wearable inertial sensors and pupillometry) can enhance the existing battery of assessment, providing more objective, natural recovery data for key subsystems (oculomotor, sensorimotor). Sex differences exist throughout development but there is a dearth of prospective research involving adolescent females who sustain a sports-related concussion and recovery. There is a need for longitudinal research in sports-related concussion as many neurophysiological deficits persist beyond the point of clinical symptom resolution (30-34).

Aims

The aim of this study is to prospectively study recovery from a sport-related concussion in adolescent male and female rugby players. The objectives of the study are to (i) examine if sensorimotor and oculomotor outcomes are affected by concussion history, sex, and age, and (ii) re-assess sensorimotor and oculomotor outcomes and self-reported measures at time points post-concussion over a 12month period.

Study design

This study comprises a cross sectional and prospective component, with follow up continuing over a single playing season. Ethical approval has been granted from Ulster University Research Ethics Committee (REC/14/0060).

Patient and Public Involvement

Participants and/or the Public WERE NOT involved in the design, or conduct, or reporting, or dissemination plans of our research.

Participants

Participants will be recruited from school or club rugby teams across the Province of Ulster. There are more than 30 school teams with male adolescent players, and 25 registered clubs with dedicated female youth teams across Ulster. Many of these sites have previously been active members of the Rugby Injury Surveillance in Ulster Schools (RISUS) and have successfully collaborated in previous studies (3, 23, 35). To be eligible for inclusion participants must be (i) aged between 16 and 18 years (ii) injury free at the time of recruitment, and (iii) currently playing rugby union in the 2022/23 playing season.

Consenting participants will complete a baseline questionnaire covering demographics characteristics (date of birth, sex, weight, height, concussion history). Parental consent is not required as the participants are aged between 16-18 and can provide their own assent for participation. The researcher team will be present throughout to assist with the understanding and completion of the questionnaire.

Methods and Analysis

Outcome measures

The outcome measures will be a range of physical assessments and self-reported questionnaires, including symptoms and psychological measures as these are often involved in sports-related concussion. All outcome measures will be taken at 3, 7, 14, 21 days and 3, 6, and 12 months post-concussive event.

Physical Assessment:

Quantified Y Balance Test is a modification of the Star Excursion Balance Test (36). A detailed testing protocol has previously published (23). Participants position themselves on a platform with hands firmly on their hips. They are instructed to use their non-stance leg to slide a box forward as far as possible with their foot and return to the starting upright position in 3 defined directions (anterior [ANT], posteromedial [PM], and posterolateral [PL]). Reach distances are recorded to the nearest 0.5 cm. Individuals complete 3 practice trials, followed by 3 recorded trials in a randomized order. A failed attempt is noted when any of the following errors occurred: touching of the foot down on the floor before returning to the starting position; placing the foot on top of the sliding box for balance; and flicking or kicking the sliding box for a better performance or any loss of balance (37). If an individual meets any of the failure criteria, the reach attempt will be discarded and repeated. During all tests, an inertial sensor (Shimmer3; Shimmer Sensing) is mounted at the level of the fourth lumbar vertebra and secured with a custom-made elastic belt to closely match the acceleration of the body's centre of mass during the Y balance test excursions. Inertial sensor testing will be captured during testing and analysed offline with MATLAB (2018b; MathWorks). Reach distances and the Y Balance test will be normalized in relation to the individuals leg length and the mean of three trials completed on each direction will be obtained. Movement control during the Y balance test will be quantified using the following variables: normalized reach distance and the SEn of the 3 axes of the gyroscope signal (x, y, and z). SEn of the signal of length N=(x1,x2,x3..xn), will be calculated using the following formula:

$$\text{Sample Entropy} = -\log (A / B)$$

Our interpretation of SEn scores is underpinned by key theories in the optimal variability during human motor performance, described by Stergiou & Decker (2011), whereby a low SEn score indicates more regular and less complex movement control during the Y balance test reach excursions, while a higher SEn score indicates a higher degree of movement irregularity/complexity (38).

For peer review only

Pupillary Light Reflex (PLR) Metrics will be measured in response to a brief, step-input, white light stimulus (154 milliseconds' duration; 180 microwatts' power) via a Neuroptics PLR-3000 handheld, infrared, automated, monocular pupillometer (Neuroptics). This device is US FDA approved and has been used in similar studies of mTBI in adults (39). The pupillometer captures dynamic responses 32 times per second, analysing a continuous, 5-second, digital video of the pupillary response to light. The metrics quantified by the device software include: maximum pupil diameter (steady-state pupil size before the light stimulus), minimum pupil diameter (pupil size after maximum constriction in response to the light stimulus), percentage pupil constriction, latency (time to maximum constriction in response to the light stimulus), peak and average constriction velocity, peak and average dilation velocity, and T75 (time for pupil redilation from minimum diameter to 75% maximum diameter). Peak dilation velocity will be calculated from automated slope-based measures obtained by the pupillometer (Microsoft Excel 2016; Microsoft Corporation).

Trained research staff will conduct PLR assessments in an environment with a room illumination of approximately 350 lux (moderate photopic viewing conditions). Athletes will focus on a 3-m distance target with the non-tested eye for ocular fixation and accommodation during a 5-second measurement period. Monocular measurements are to be repeated at least 3 times for each eye, alternating 1-minute time intervals to allow rapid visual light adaptation, to obtain 2 to 3 artifact-free responses per eye. The combined mean of each pupillometry metric is then calculated for at least 2 assessments without artifacts, defined as blinks or eye movements occurring within the first 3 seconds of the response.

Self-report questionnaires:

The Post-Concussion Symptom Scale (PCSS) consists of 22 questions that relate to post-concussive symptoms. Users are asked to rate each symptom according to a 7-point Likert scale ranging from 0-6. Higher scores indicate a higher severity of post-concussive symptoms. The greatest possible score is 132 and the lowest possible score is 0. The scale has a high internal consistency (40) in healthy and concussed adolescents, its construct validity has been established (41) and it can accurately discriminate between concussed and non-concussed athletes (40).

The Paediatric Fear Avoidance Behaviour after Traumatic Brain Injury Questionnaire (PFAB-TBI) is a 16-item measure designed to assess fear avoidance following paediatric concussion developed based on FAB-TBI. The FAB-TBI was developed to assess fear avoidance behaviour post-concussion in adults. Items are related to avoidance of activities (e.g. work) due to fear of symptom consequences or symptom exacerbation (e.g. worsened headache). For the PFAB-TBI self-report item content will remain the same with the exception of two items. Item four will be changed from "My work might harm my brain" to "My schoolwork might hurt my brain" and item five will be changed from, "I should not do my normal work with my present symptoms" to "I should not do my normal schoolwork with my present symptoms." The PFAB– TBI asks respondents to rate agreement on statements related to avoidance behaviour over the past month from 0 (strongly disagree) to 3 (strongly agree). A total sum score will be used in the current study, where higher scores indicate greater fear avoidance behaviour (possible range 0–48). Change scores will be calculated across testing dates (29).

For peer review only

The general anxiety disorder (GAD-7) is a seven-item questionnaire that has questions about possible problems the respondent may have been bothered by over the previous 2 weeks. Each question has four possible responses from “not at all” to “nearly every day” and scored 0 to 3 respectively. The GAD-7 has reported good reliability, as well as criterion, construct, factorial, and procedural validity (42).

The Patient Health Questionnaire (PHQ-9) is a self-reported questionnaire with nine questions about experienced depression symptoms. Questions cover interest in things, feelings of hopelessness, sleep, fatigue, appetite, concentration and thoughts about suicide. Each question is scored from a "0" (not at all) to "3" (nearly every day) and has been validated for primary care use (43).

The Sport-related concussion clinical profiling questionnaire consisting of 29 questions covering possible symptoms and severity of symptoms a player may have experienced over the past 24 hours. Each question has a four-point scale ranging from no symptoms experienced to severe, and scored 0 – 3 for each question. The questionnaire explores five possible clinical profiles including cognitive/fatigue, vestibular, ocular, migraine, and anxiety/mood and two modifiers, sleep, and neck pain (44).

Sport-related Concussion events

Sport-related concussion prevalence will be recorded over a single playing season (2022/23). Sport-related concussion definition will follow the criteria set out by the Zurich Consensus Statement on Concussion in Sport (1); i.e. a direct blow to the head, face, neck or elsewhere on the body with an impulsive force transmitted to the head resulting in one or more of the symptoms in one or more of the following clinical domains (which may or may not have involved loss of consciousness): somatic, cognitive, emotional/behavioural, and sleep disturbance. All sport-related concussions must be verified by a medical professional within 48 hours of the injury. When a participant is diagnosed with a concussion, the designated person at each school/club (data champion), will be responsible for contacting the primary researcher (CMcK). CMcK will maintain weekly contact with the data champion, sending study reminders via text message or a follow-up phone call. The following information will be inputted for each concussion: date of injury, reporter/person making the diagnosis, site where diagnosis was made, cause of injury (phase of play, direct vs indirect), time to first symptoms, nature of first symptoms.

In the event of a sport-related concussion, all outcome measures will be reassessed by the primary researcher after 3, 7, 14 and 21 days, and 3, 6 and 12 months. Dates of return to training and return to play will be self-reported by the participant or coach. Return to play dates will be cross referenced with the official team lists registered with Ulster Rugby. The researchers involved in this study will have no influence on the return to training or return to play decisions; these decisions will remain external to the current project. Participants will be asked on return visits as to whether they have experienced any treatment or intervention relating to their concussion between visits. Any and all interventions undertaken by individuals will be considered and reported when analysing the results.

Statistical Analysis

For peer review only

All data will be analysed using the Statistical Package of Social Sciences (SPSS) (Version 26; SPSS Inc).

Baseline outcomes (Concussion history, Y Balance, PLRM) will be summarized using means and standard deviations for scale variables and frequencies and percentages for nominal variables. We will use histograms to examine the frequency distributions of scale variables, and where normality is assumed, we will use independent t-tests to compare baseline data (PCSS, Y Balance, PLRM), with players categorised into two groups based on self-reported history of concussion in the last 12 months (no concussion in the last 12 months or concussion within the last 12 months). To avoid bias, researchers will be blinded to group title (i.e. history of concussion v no history of concussion). The threshold for statistical significance will $P < .01$ for all tests. We will also present mean differences (95% confidence intervals) between groups for scale outcome data.

Sport-related concussions occurring throughout the 2022/23 playing season will be summarized using counts (n, %). Time to sport-related concussion event will be plotted using survival curves, with participants censored at the end of the playing season. Serial outcome data (PCSS, Y Balance, PLRM, PFAB-TBI) will be measured at 72 hours, 7 days, 14 days, 23 days, 90 days, 180 days and 365 days post-concussion; we will initially determine the number (%) of participants that had returned to baseline at each time point. We will also examine the growth/response curves for each participant, as well as compare curves between known groups; sex and concussion history. As the shape of the time-response curve is uncertain, and may vary across each outcome variable, we will calculate summary measures for each participant based on either the peak value, the time to return to baseline, or the area under the curve. Area under the curve will be calculated using the trapezium rule (by adding the areas under the graph between each pair of consecutive observations). The most suitable summary measure will be used in the calculation of a median score (interquartile ranges) for each outcome variable. Between group comparisons will also be made, comparing curves across two known groups e.g., males/females and history of concussion/no history.

In an exploratory analysis, we will use logistic regression to examine the association between demographic variables (age, sex, previous injury) and recovery at 23 day follow up. We will run separate models for each outcome measure (PCSS, Y Balance, PLRM, PFAB-TBI). Our previous research (3) found that 90% of adolescent rugby players return to sport at 23 days post-concussion; this is also the minimal convalesce for adolescent players post-concussion based on Irish Rugby Football Union and World Rugby guidance, which are built around the latest medical research and expert based opinion (45). Full recovery will be defined as a return to baseline (pre-season) values for each respective outcome. Initially we will conduct a series of univariate analyses to determine whether any predictor variables are associated with recovery. Correlations among predictor variables will be calculated to screen for any strong co-linearity ($r>0.8$). Predictors demonstrating a p-value less than 0.10 on univariate testing will be entered into a multiple logistic regression analysis. The strength of the predictive model will be determined using R^2, p-values, odds ratios, with 95% confidence intervals (95% CI).

Sample size

For peer review only

We aim to enrol 250 participants at baseline. This figure allows for a 20% loss to follow-up over the entire study and assumes a 10% concussion prevalence over a single playing season. This will generate recovery data from approximately 20 participants presenting with a new concussion. Recruitment will be stratified to include equal numbers of male and female participants (n=125 male; n=125 female).

A sample of 250 participants would also provide sufficient power for detect baseline difference in sensorimotor function. Our previous research (23) had a medium effect difference SMD 0.5 between adolescents with a history of concussion to those with no history for sensorimotor function with standard deviation of 0.22 units (SEn). Based on an alpha level of 1%, n=180 participants would provide 90% power for detecting a medium effect difference in SEn between groups at baseline.

Discussion

The primary aim of this study is to study recovery from a sport-related concussion in adolescent male and female rugby players and whether this is affected by concussion history, sex, and age. The ability to confidently diagnose a concussion and monitor its resolution in adolescents has proven somewhat challenging. For an initial sport-related concussion diagnosis in adolescents, no single, gold-standard test exists (46), and even widely-accepted tests have questionable reliability, such as the mBESS, with which groups have been found to have high variability and large number of errors (47). Multiple areas of SRC sign and symptom endorsement should be evaluated, which requires an individualized approach to each athlete and injury (46). Given the absence of a diagnostic test or biomarker for concussion in adolescents, the current cornerstone of concussion diagnosis is confirming the presence of a constellation of signs and symptoms with multiple tests after an individual has experienced a hit to the head or body although existing evidence is insufficient to determine the best combination of measures (48).

A mix of quantitative and qualitative metrics have been chosen to identify and track recovery from concussion including several self-report measures. Despite the documented importance of reporting concussive symptoms, research has suggested that up to half of concussions in adolescent football players are unreported and therefor undiagnosed (49). The mixed-methods approach employed in the proposed research may allow for an interesting view of whether adolescent rugby players under-report symptoms when quantitatively measured metrics are still elevated.

Women’s rugby is growing in popularity but there is little evidence available regarding injuries and in particular recovery from concussion (50). Research has highlighted that they experience worse outcomes associated with concussion (51) and are more likely to experience a concussion when compared with males (52) and this highlights an area of focus for our research.

This trial protocol aims to optimise methodological quality and report pragmatic clinical findings by addressing key methodological limitations of other studies that have aimed to track recovery of concussion in adolescents. Key strengths of the study include: (1) a large even sample population of adolescent male and female rugby players, (2) participant

stratification into pre-determined subgroups based on predetermined criteria (i.e. concussion history), (3) multiple outcome measures to assess a wide array of deficits associated with sport-related concussion and (4) longitudinal follow-up to explore possible persistence of multi-system deficits associated with SRC.

LIST OF ABBREVIATIONS

SCAT-Sport Concussion Assessment Tool

PCSS-Post-Concussion Symptom Scale

SAC-Standardised Assessment of Concussion

m-BESS-Modified Balance Error Scoring System

PLR-Pupillary Light Response

PFAB-TBI-Paediatric Fear Avoidance Behaviour after Traumatic Brain Injury

Ethics and Dissemination

Ethics Approval and Consent to Participate

Participating in this study poses little to no risk to participants or their parents. All patients and their parents/guardians will provide written informed consent/assent and will have the ability to withdraw at any time without explanation. Participation in the study will not restrict them from receiving the standard diagnosis and management, as determined by treating physicians. We will ensure that data remain secure, and that patient privacy and confidentiality are maintained.

Ethical approval has been granted through the Ulster University ethics committee (REC/14/0060).

Dissemination

The findings of this research will be published in an peer-reviewed open-access medical journal. All positive, inconclusive or negative findings will be disseminated to maximise the social value of the research and to accurately inform future practises. This will occur as soon as possible after completion of the final analysis.

Consent for Publication

N/A

Availability for Data and Materials

All data generated or analysed during this study will be included in this published article [and its supplementary information files].

For peer review

Competing Interests

The authors declare that they have no competing interests.

Funding

This research is to be completed during a PhD funded by the Department for the Economy at Ulster University. These provide for the payments of approved fees and the maintenance of students whilst being trained in methods of PhD research- as such there is no specific award/grant number for this project from my funder that I can provide. The funding body will have no impact on the study design, data collection, analysis, interpretation of data or in writing the manuscript.

Author Contributions- Contributorship Statement

All authors contributed to the concept, design and writing of this protocol.

CM, MM, AR, CB each had substantial contributions to the conception, design, planning, as well as the acquisition, analysis and interpretation of data for the work. CM drafted the initial content, and MM, AR and CB reviewed it critically for important intellectual content, suggesting and making changes to the work to ensure the highest quality. CM, MM, AR and CB collectively approved the final version to be published. CM, MM, AR and CB were in agreement to be accountable for all aspects of work in ensuring that questions related to the accuracy or integrity of any part of the work were appropriately investigated and resolved.

Acknowledgements

Not applicable.

Authors' Information

N/A

Footnotes

N/A

For peer review only

References

1. McCrory P, Meeuwisse W, Dvořák J, Aubry M, Bailes J, Broglio S, et al. Consensus statement on concussion in sport-the 5(th) international conference on concussion in sport held in Berlin, October 2016. British journal of sports medicine. 2017;51(11):838-47.
2. Macpherson A, Fridman L, Scolnik M, Corallo A, Guttmann A. A population-based study of paediatric emergency department and office visits for concussions from 2003 to 2010. (1205-7088 (Print)).
3. Archbold HA, Rankin AT, Webb M, Nicholas R, Eames NW, Wilson RK, et al. RISUS study: Rugby Injury Surveillance in Ulster Schools. British journal of sports medicine. 2017;51(7):600-6.
4. Lawrence JB, Haider MN, Leddy JJ, Hinds A, Miecznikowski JC, Willer BS. The King-Devick test in an outpatient concussion clinic: Assessing the diagnostic and prognostic value of a vision test in conjunction with exercise testing among acutely concussed adolescents. Journal of the neurological sciences. 2019;398:91-7.
5. Corbin-Berrigan L-A, Gagnon I. Postconcussion Symptoms as a Marker of Delayed Recovery in Children and Youth Who Recently Sustained a Concussion: A Brief Report. Clinical journal of sport medicine. 2017;27(3):325-7.
6. Berkner J, Meehan WP, 3rd, Master CL, Howell DR. Gait and Quiet-Stance Performance Among Adolescents After Concussion-Symptom Resolution. Journal of athletic training. 2017;52(12):1089-95.
7. Caze T, Vásquez D, Moffatt K, Waple K, Hope D. A Prospective Pilot Study of Anxiety Sensitivity and Adolescent Sports-Related Concussion. Archives of clinical neuropsychology. 2020;36(6):930-9.
8. Rhine T, Babcock L, Zhang N, Leach J, Wade SL. Are UCH-L1 and GFAP promising biomarkers for children with mild traumatic brain injury? Brain injury. 2016;30(10):1231-8.
9. Davis GA, Anderson V, Babl FE, Gioia GA, Giza CC, Meehan W, et al. What is the difference in concussion management in children as compared with adults? A systematic review. British journal of sports medicine. 2017;51(12):949-57.
10. Guskiewicz KM, Register-Mihalik JK. Postconcussive impairment differences across a multifaceted concussion assessment protocol. PM & R : the journal of injury, function, and rehabilitation. 2011;3(10 Suppl 2):445.
11. Harmon KG, Clugston JR, Dec K, Hainline B, Herring S, Kane SF, et al. American Medical Society for Sports Medicine position statement on concussion in sport. British journal of sports medicine. 2019;53(4):213-25.
12. Rose SC, Fischer AN, Heyer GL. How long is too long? The lack of consensus regarding the post-concussion syndrome diagnosis. Brain injury. 2015;29(7-8):798-803.
13. Echemendia RJ, Meeuwisse W, McCrory P, Davis GA, Putukian M, Leddy J, et al. The Sport Concussion Assessment Tool 5th Edition (SCAT5): Background and rationale. British journal of sports medicine. 2017;51(11):848-50.
14. Howell DR, Brien MJ, Beasley MA, Mannix RC, Meehan Iii WP. Initial somatic symptoms are associated with prolonged symptom duration following concussion in adolescents. Acta Paediatrica. 2016;105(9):e426-e32.
15. Hang B, Babcock L, Hornung R, Ho M, Pomerantz W. Can Computerized Neuropsychological Testing in the Emergency Department Predict Recovery for Young Athletes With Concussions? Pediatric emergency care. 2015;31(10):688-93.
16. Lau B, Lovell M, Collins M, Pardini J. Neurocognitive and Symptom Predictors of Recovery in High School Athletes. Clinical journal of sport medicine. 2009;19(3):216-21.

For peer review only

17. Ellis MJ, Cordingley DM, Vis S, Reimer KM, Leiter J, Russell K. Clinical predictors of vestibulo-ocular dysfunction in pediatric sports-related concussion. Journal of Neurosurgery Publishing Group (JNSPG); 2017. p. 38.
18. Master C, Master S, Wiebe D, Storey E, Lockyer J, Podolak O, et al. Vision and Vestibular System Dysfunction Predicts Prolonged Concussion Recovery in Children. Clinical journal of sport medicine. 2018;28(2):139-45.
19. Ellis MJ, Cordingley D, Vis S, Reimer K, Leiter J, Russell K. Vestibulo-ocular dysfunction in pediatric sports-related concussion. Journal of Neurosurgery Publishing Group (JNSPG); 2015. p. 248.
20. Powers KC, Kalmar JM, Cinelli ME. Recovery of static stability following a concussion. Gait & posture. 2014;39(1):611-4.
21. Broglio SP, Macciocchi SN, Ferrara MS. Sensitivity of the concussion assessment battery. Neurosurgery. 2007;60(6):1050-8.
22. Johnston W, O'Reilly M, Duignan C, Liston M, McLoughlin R, Coughlan GF, et al. Association of Dynamic Balance With Sports-Related Concussion: A Prospective Cohort Study. The American Journal of Sports Medicine. 2019;47(1):197-205.
23. Matthews M, Johnston W, Bleakley CM, Davies RJ, Rankin AT, Webb M, et al. Concussion History and Balance Performance in Adolescent Rugby Union Players. The American Journal of Sports Medicine. 2021;49(5):1348-54.
24. Master CL, Podolak OE, Ciuffreda KJ, Metzger KB, Joshi NR, McDonald CC, et al. Utility of Pupillary Light Reflex Metrics as a Physiologic Biomarker for Adolescent Sport-Related Concussion. JAMA ophthalmology. 2020;138(11):1135-41.
25. Boev AN, Fountas KN, Karampelas I, Boev C, Machinis TG, Feltes C, et al. Quantitative pupillometry: normative data in healthy pediatric volunteers. Journal of neurosurgery. 2005;103(6 Suppl):496-500.
26. Woodrome SE, Yeates KO, Taylor HG, Rusin J, Bangert B, Dietrich A, et al. Coping strategies as a predictor of post-concussive symptoms in children with mild traumatic brain injury versus mild orthopedic injury. Journal of the International Neuropsychological Society : JINS. 2011;17(2):317-26.
27. Silverberg ND, Panenka WJ, Iverson GL. Fear Avoidance and Clinical Outcomes from Mild Traumatic Brain Injury. Journal of neurotrauma. 2018;35(16):1864-73.
28. Wijenberg MLM, Stapert SZ, Verbunt JA, Ponsford JL, Van Heugten CM. Does the fear avoidance model explain persistent symptoms after traumatic brain injury? Brain injury. 2017;31(12):1597-604.
29. Cairncross M, Brooks BL, Virani S, Silverberg ND. Fear avoidance behavior in youth with poor recovery from concussion: measurement properties and correlates of a new scale. Child neuropsychology : a journal on normal and abnormal development in childhood and adolescence. 2021;27(7):911-21.
30. Mutch WAC, Ellis MJ, Ryner LN, Morissette MP, Pries PJ, Dufault B, et al. Longitudinal Brain Magnetic Resonance Imaging CO2 Stress Testing in Individual Adolescent Sports-Related Concussion Patients: A Pilot Study. Frontiers in neurology. 2016;7:107.
31. McGuine TA, Pfaller A, Hetzel S, Broglio SP, Hammer E. A Prospective Study of Concussions and Health Outcomes in High School Football Players. Journal of athletic training. 2020;55(10):1013-9.
32. Howell D, Myer G, Brilliant A, Barber Foss K, Meehan W. Quantitative Multimodal Assessment of Concussion Recovery in Youth Athletes. Clinical journal of sport medicine. 2019;31(2):133-8.
33. Nolin P, Stipanicic A, Henry M, Joyal CC, Allain P. Virtual reality as a screening tool for sports concussion in adolescents. Brain injury. 2012;26(13-14):1564-73.

For peer review only

34. Kelty-Stephen DG, Qureshi Ahmad M, Stirling L. Use of a Tracing Task to Assess Visuomotor Performance for Evidence of Concussion and Recuperation. Psychological assessment. 2015;27(4):1379-87.
35. Archbold P, Rankin A, Webb M, Davies R, Nicholas R, Eames N, et al. Injury patterns in U15 rugby players in Ulster Schools: A Rugby Injury Surveillance (RISUS) study. Translational Sports Medicine. 2021;4.
36. Shaffer SW, Teyhen DS, Lorenson CL, Warren RL, Koreerat CM, Straseske CA, et al. Y-balance test: a reliability study involving multiple raters. Military medicine. 2013;178(11):1264-70.
37. Gribble PA, Hertel J, Plisky P. Using the Star Excursion Balance Test to assess dynamic postural-control deficits and outcomes in lower extremity injury: a literature and systematic review. Journal of athletic training. 2012;47(3):339-57.
38. Stergiou N, Decker LM. Human movement variability, nonlinear dynamics, and pathology: is there a connection? (1872-7646 (Electronic)).
39. Ciuffreda KJ, Joshi NR, Truong JQ. Understanding the effects of mild traumatic brain injury on the pupillary light reflex. Concussion (London, England). 2017;2(3):CNC36-0029. eCollection 2017 Nov.
40. Lovell MR, Iverson GL, Collins MW, Podell K, Johnston KM, Pardini D, et al. Measurement of symptoms following sports-related concussion: reliability and normative data for the post-concussion scale. Applied Neuropsychology. 2006;13(3):166-74.
41. Riegler KE, Guty ET, Arnett PA. Validity of the ImPACT Post-Concussion Symptom Scale (PCSS) Affective Symptom Cluster as a Screener for Depression in Collegiate Athletes. Archives of clinical neuropsychology : the official journal of the National Academy of Neuropsychologists. 2019;34(4):563-74.
42. Spitzer RL, Kroenke K, Williams JBW, Löwe B. A Brief Measure for Assessing Generalized Anxiety Disorder: The GAD-7. Archives of Internal Medicine. 2006;166(10):1092-7.
43. Cameron IM, Crawford JR, Lawton K, Reid IC. Psychometric comparison of PHQ-9 and HADS for measuring depression severity in primary care. The British journal of general practice : the journal of the Royal College of General Practitioners. 2008;58(546):32-6.
44. Kontos A, Trbovich A, Sandel N, Emami K, Collins M. Sport-related Concussion Clinical Profiles: Clinical Characteristics, Targeted Treatments, and Preliminary Evidence. Current Sports Medicine Reports. 2019;18:82-92.
45. Irfu. IRFU Return to play Protocol. 2022.
46. McKeithan L, Hibshman N, Yengo-Kahn AM, Solomon GS, Zuckerman SL. Sport-Related Concussion: Evaluation, Treatment, and Future Directions. Medical sciences (Basel, Switzerland). 2019;7(3):44. doi: 10.3390/medsci7030044.
47. Starling AJ, Leong DF, Bogle JM, Vargas BB. Variability of the modified Balance Error Scoring System at baseline using objective and subjective balance measures. Concussion (London, England). 2015;1(1):CNC5.
48. Giza CC, Kutcher JS, Ashwal S, Barth J, Getchius TS, Gioia GA, et al. Summary of evidence-based guideline update: evaluation and management of concussion in sports: report of the Guideline Development Subcommittee of the American Academy of Neurology. Neurology. 2013;80(24):2250-7.
49. McCrea M, Hammeke T, Olsen G, Leo P, Guskiewicz K. Unreported concussion in high school football players: implications for prevention. Clinical journal of sport medicine : official journal of the Canadian Academy of Sport Medicine. 2004;14(1):13-7.
50. Moody JR, Feiss RS, Pangelinan MM. A systematic review of acute concussion assessment selection in research. Brain injury. 2019;33(8):967-73.

For peer review only

51. Baker JG, Leddy JJ, Darling SR, Shucard J, Makdissi M, Willer BS. Gender Differences in Recovery From Sports-Related Concussion in Adolescents. Clinical pediatrics. 2016;55(8):771-5.
52. Mollayeva T, El-Khechen-Richandi G, Colantonio A. Sex & gender considerations in concussion research. Concussion (London, England). 2018;3(1):CNC51-0015. eCollection 2018 Mar.

For peer review only

STROBE Statement—checklist of items that should be included in reports of observational studies

	Item No	Recommendation	Page No
**Title and abstract**	1	(*a*) Indicate the study's design with a commonly used term in the title or the abstract	1
		(*b*) Provide in the abstract an informative and balanced summary of what was done and what was found	1
**Introduction**			
Background/rationale	2	Explain the scientific background and rationale for the investigation being reported	2-4
Objectives	3	State specific objectives, including any prespecified hypotheses	4
**Methods**			
Study design	4	Present key elements of study design early in the paper	4
Setting	5	Describe the setting, locations, and relevant dates, including periods of recruitment, exposure, follow-up, and data collection	4-5
Participants	6	(*a*) *Cohort study*—Give the eligibility criteria, and the sources and methods of selection of participants. Describe methods of follow-up<br>*Case-control study*—Give the eligibility criteria, and the sources and methods of case ascertainment and control selection. Give the rationale for the choice of cases and controls<br>*Cross-sectional study*—Give the eligibility criteria, and the sources and methods of selection of participants	4-8
		(*b*) *Cohort study*—For matched studies, give matching criteria and number of exposed and unexposed<br>*Case-control study*—For matched studies, give matching criteria and the number of controls per case	n/a
Variables	7	Clearly define all outcomes, exposures, predictors, potential confounders, and effect modifiers. Give diagnostic criteria, if applicable	5-9
Data sources/ measurement	8*	For each variable of interest, give sources of data and details of methods of assessment (measurement). Describe comparability of assessment methods if there is more than one group	5-8
Bias	9	Describe any efforts to address potential sources of bias	8
Study size	10	Explain how the study size was arrived at	8-9
Quantitative variables	11	Explain how quantitative variables were handled in the analyses. If applicable, describe which groupings were chosen and why	7-8
Statistical methods	12	(*a*) Describe all statistical methods, including those used to control for confounding	7-9
		(*b*) Describe any methods used to examine subgroups and interactions	7-8
		(*c*) Explain how missing data were addressed	n/a
		(*d*) *Cohort study*—If applicable, explain how loss to follow-up was addressed	8-9

For peer review only

		Case-control study—If applicable, explain how matching of cases and controls was addressed<br>*Cross-sectional study*—If applicable, describe analytical methods taking account of sampling strategy	
		(*e*) Describe any sensitivity analyses	8-9
**Results**			
Participants	13*	(a) Report numbers of individuals at each stage of study—eg numbers potentially eligible, examined for eligibility, confirmed eligible, included in the study, completing follow-up, and analysed	8-9
		(b) Give reasons for non-participation at each stage	n/a
		(c) Consider use of a flow diagram	n/a
Descriptive data	14*	(a) Give characteristics of study participants (eg demographic, clinical, social) and information on exposures and potential confounders	4-5
		(b) Indicate number of participants with missing data for each variable of interest	n/a
		(c) *Cohort study*—Summarise follow-up time (eg, average and total amount)	7
Outcome data	15*	*Cohort study*—Report numbers of outcome events or summary measures over time	7
		Case-control study—Report numbers in each exposure category, or summary measures of exposure	n/a
		Cross-sectional study—Report numbers of outcome events or summary measures	*n/a*
Main results	16	(*a*) Give unadjusted estimates and, if applicable, confounder-adjusted estimates and their precision (eg, 95% confidence interval). Make clear which confounders were adjusted for and why they were included	7-8
		(*b*) Report category boundaries when continuous variables were categorized	7-8
		(*c*) If relevant, consider translating estimates of relative risk into absolute risk for a meaningful time period	n/a
Other analyses	17	Report other analyses done—eg analyses of subgroups and interactions, and sensitivity analyses	7-8
**Discussion**			
Key results	18	Summarise key results with reference to study objectives	9-10
Limitations	19	Discuss limitations of the study, taking into account sources of potential bias or imprecision. Discuss both direction and magnitude of any potential bias	10
Interpretation	20	Give a cautious overall interpretation of results considering objectives, limitations, multiplicity of analyses, results from similar studies, and other relevant evidence	9-10
Generalisability	21	Discuss the generalisability (external validity) of the study results	9-10
**Other information**			
Funding	22	Give the source of funding and the role of the funders for the present study and, if applicable, for the original study on which the present article is based	11

Continued on next page

For peer review only - http://bmjopen.bmj.com/site/about/guidelines.xhtml

*Give information separately for cases and controls in case-control studies and, if applicable, for exposed and unexposed groups in cohort and cross-sectional studies.

Note: An Explanation and Elaboration article discusses each checklist item and gives methodological background and published examples of transparent reporting. The STROBE checklist is best used in conjunction with this article (freely available on the Web sites of PLoS Medicine at http://www.plosmedicine.org/, Annals of Internal Medicine at http://www.annals.org/, and Epidemiology at http://www.epidem.com/). Information on the STROBE Initiative is available at www.strobe-statement.org.

For peer review only

For peer review only - http://bmjopen.bmj.com/site/about/guidelines.xhtml
